# Combined CCTA and Stress CTP for Anatomical and Functional Assessment of Myocardial Bridges

**DOI:** 10.3390/jimaging11090324

**Published:** 2025-09-21

**Authors:** Marco Fogante, Paolo Esposto Pirani, Fatjon Cela, Enrico Paolini, Liliana Balardi, Nicolò Schicchi

**Affiliations:** 1 Maternal-Child, Senological, Cardiological Radiology and Outpatient Ultrasound, Department of Radiological Sciences, University Hospital of Marche, 60126 Ancona, Italy; 2 Cardiology and Intensive Cardiac Care Unit, Cardiovascular Department, University Hospital of Marche, 60126 Ancona, Italy; 3 Health Professions Area, Diagnostic Technical Area, University Hospital of Marche, 60126 Ancona, Italy; liliana.balardi@ospedaliriuniti.marche.it; 4 Cardiovascular Radiological Diagnostics, Department of Radiological Sciences, University Hospital of Marche, 60126 Ancona, Italy

**Keywords:** myocardial bridging, coronary computed tomography angiography, CT myocardial perfusion imaging, myocardial ischemia, coronary artery disease

## Abstract

Myocardial bridging (MB) is a congenital coronary anomaly whose clinical impact remains controversial. Coronary computed tomography angiography (CCTA) combined with CT myocardial perfusion imaging (CT-MPI) enables a comprehensive anatomical and functional assessment of MB. This study aimed to investigate whether specific high-risk anatomical features of MB are independently associated with myocardial hypoperfusion, using combined CCTA and CT-MPI. We retrospectively analyzed 81 patients with MB showing high-risk anatomical features (depth ≥ 2.0 mm and length ≥ 25 mm) identified by CCTA, all of whom underwent stress dynamic CT-MPI between May 2022 and December 2025. Patients were classified according to the presence or absence of hypoperfusion in MB-related myocardial segments. Clinical and anatomical variables were compared between two groups using non-parametric tests, and multivariable logistic regression was performed to identify independent predictors of hypoperfusion. Among the 81 patients (mean age, 59.3 ± 11.7 years; 54 males), 26 (32.1%) demonstrated perfusion defects. All MBs were located in the left anterior descending artery (LAD). No significant differences were observed in clinical variables between groups. Bridges associated with hypoperfusion were significantly deeper (*p* < 0.001) and were more frequently located in the mid-LAD (73.1% vs. 38.2%, *p* = 0.01). In multivariable analysis, bridge depth and mid-LAD location remained independent predictors of hypoperfusion. In patients with MB, greater depth and mid-LAD location are independently associated with myocardial hypoperfusion. The combined use of CCTA and CT-MPI may enhance risk stratification and help guide clinical decision-making in this patient population.

## 1. Introduction

Myocardial bridging (MB) is a congenital coronary anomaly in which a portion of an epicardial artery follows a partially or completely intramyocardial course [1]. Although traditionally considered benign, MB may become clinically relevant, particularly during tachycardia, when altered systolic/diastolic flow ratio may lead to myocardial ischemia, arrhythmias, or even sudden cardiac death [2]. The depth and length of MB, when exceeding 2.0 mm and 25 mm, respectively, are considered high-risk anatomical factors associated with increased hemodynamic significance and potential ischemic symptoms in selected cases [3]. However, emerging evidence suggests that MB is not merely an anatomical but a dynamic condition, with its clinical impact influenced by both morphological features and functional mechanisms, such as endothelial dysfunction, microvascular dysregulation, and altered shear stress, making its functional significance a subject of ongoing debate [4,5].

The use of coronary computed tomography angiography (CCTA) has significantly improved the detection and the anatomical evaluation of MBs. Depending on the CT scanner generation, patient population, and MB classification criteria, CT-based prevalence estimates range widely, from approximately 5.7% to 58% [6]. However, CCTA provides only anatomical information, and in many cases, especially when MBs are incidentally detected, their functional significance remains unclear [7]. Functional assessment traditionally relies on single photon emission computed tomography (SPECT) or invasive fractional flow reserve (FFR) or acetylcholine testing, which are often time-consuming, radiation-intensive, or limited to specialized centers [8,9]. In this context, stress dynamic CT-myocardial perfusion imaging (MPI) has emerged as a valuable technique for assessing cardiac perfusion [10,11]. CT-MPI has shown diagnostic accuracy comparable to that of SPECT with additional advantages in terms of speed, patient comfort, and radiation dose [12,13].

Despite the availability of these tools, limited data exist on the possible association between anatomical factors of MB and objective evidence of perfusion impairment on CT-MPI [14,15]. To our knowledge, no prior study has focused exclusively on high-risk MBs using this combined approach in a homogeneous patient cohort.

The aim of this study was to investigate whether specific high-risk anatomical features of MB are independently associated with myocardial hypoperfusion, using combined CCTA and CT-MPI.

## 2. Materials and Methods

### 2.1. Study Population

**Chart 1:**
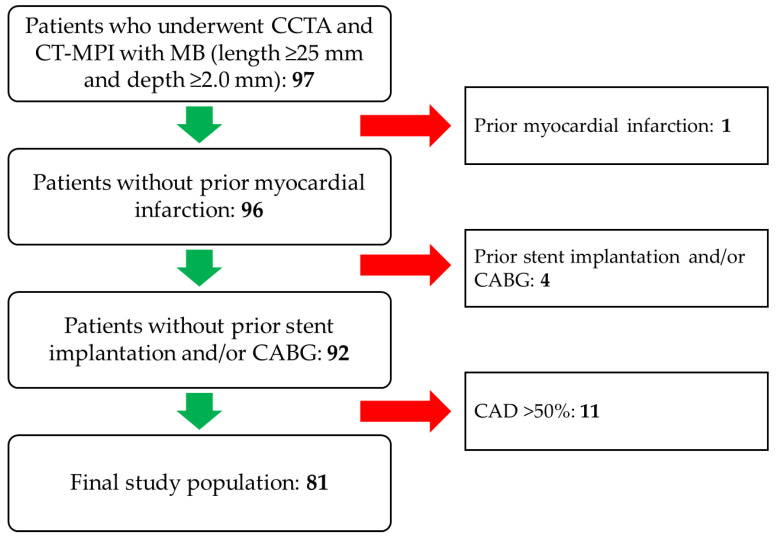
Study population selection. Abbreviations—CCTA: coronary computed tomography angiography; CT-MPI: computed tomography-myocardial perfusion imaging; MB: myocardial bridging; CABG: coronary artery bypass graft; CAD: coronary artery disease.

This retrospective study enrolled patients identified from the Radiological Database of our Institution who underwent CCTA combined with stress dynamic CT-MPI between May 2022 and December 2025 due to clinical suspicion of coronary artery disease. Patients were screened for inclusion based on the presence of an MB on any of the three major coronary arteries. Eligible bridges were required to meet predefined high-risk anatomical criteria, specifically a length ≥ 25 mm and a depth ≥ 2.0 mm. To ensure a homogenous study population and isolate the functional impact of MB, patients were excluded if they had coronary artery stenosis >50%, previous coronary stent implantation or coronary artery bypass grafting (CABG), or a prior history of myocardial infarction. Out of an initial cohort of 97 patients, 16 were excluded due to the following reasons: history of myocardial infarction (*n* = 1); prior stent placement or CABG surgery (*n* = 4); coronary artery stenosis >50% (*n* = 11). Thus, 81 patients were included in the final analysis (Chart 1). For each patient included in the study, clinical data were retrospectively collected to support correlation with imaging findings. The study was conducted in accordance with institutional ethical standards, with approval obtained from the local Ethics Committee (ID: 3560; approved on 20 February 2025). Written informed consent was obtained from all participants prior to inclusion.

### 2.2. Scan Protocol

All examinations were conducted using a third-generation dual-source CT system equipped with 384 slices (2 × 192 detectors) (SOMATOM Force; Siemens Healthineers; Germany). Patients were instructed to refrain from caffeine intake for at least 24 h prior to the scan. A 20-gauge intravenous cannula was placed in the right antecubital vein and connected to a dual-head power injector. The imaging protocol was standardized and included three sequential phases. Initially, a non-contrast electrocardiogram (ECG)-triggered scan was performed to define the anatomical scan range for dynamic CTP. CCTA was then acquired using an ECG-gated protocol. Contrast enhancement for CCTA was achieved using 40 mL of iopamidol (370 mg I/mL) injected at 5.5 mL/s. Stress dynamic CT-MPI followed, initiated one minute after the administration of 5 mL of regadenoson over 10 s, followed by a 10 mL saline flush. Subsequently, 60 mL of contrast medium was injected at 6 mL/s, followed by a 40 mL saline chaser. Dynamic image acquisition used a shuttle-mode technique, allowing multiphase coverage over approximately 30 s. Depending on the heart rate, between 15 and 20 perfusion phases were obtained. Throughout the exam, patients’ cardiac rhythm was continuously monitored, and blood pressure was recorded before and after stress testing. Automatic tube voltage selection (CAREkV, Siemens Healthineers, Forchheim, Germany) and dose modulation (CAREDose4D, Siemens Healthineers, Forchheim, Germany) were applied in all acquisitions. Image reconstruction was performed using an iterative algorithm (Advanced Modeled Iterative Reconstruction, Siemens Healthineers, Forchheim, Germany) with strength level 4.

### 2.3. CCTA Analysis

CCTA images were analyzed using dedicated software (syngo.via Cardiac CT, version VB10A, Siemens Healthineers, Forchheim, Germany). Two sets of axial images were reconstructed applying both a smooth kernel (Body vascular 40) and a sharp kernel (Body vascular 44) to optimize visualization of coronary plaques and vessel wall morphology. Coronary artery evaluation included the assessment of coronary artery calcium score (CACS), the anatomical characteristics of MBs and the presence and severity of coronary stenosis. CACS was performed on non-contrast scans using the Agatston method. MBs were systematically evaluated in terms of their anatomical location, tunneled segment length (in mm), and maximum depth (in mm). Coronary stenoses were visually classified according to the CAD-RADS 2.0 system, and stenoses in the vessel segment containing the MB were specifically noted. Epicardial adipose tissue (EAT) volume was quantified using the non-contrast images. The fibrous pericardium was manually delineated on axial images at 10 mm intervals, starting from the level of the pulmonary artery bifurcation down to the diaphragm. Fat tissue was identified by applying a Hounsfield Unit range between −190 and −30. The total EAT volume, expressed in cm^3^, corresponded to the amount of fat included within the manually traced pericardial contour, and was automatically computed through 3D reconstruction. All image analyses were independently performed by two cardiovascular radiologists, each with over 10 years of experience in cardiac CT. Coronary artery segmentation followed the 17-segment model of the American Heart Association. The readers were blinded to each other’s assessments and to all clinical and perfusion data. In the event of disagreement, a consensus reading was used to determine the final interpretation.

### 2.4. Stress Dynamic CT-MPI Analysis

Stress dynamic CTP images were analyzed using a dedicated clinical software (syngo.via Myocardial Perfusion Analysis, version VB10A, Siemens Healthineers, Forchheim, Germany). Myocardial blood flow (MBF) was assessed according to a 17-segment model, excluding the apex. For septal and perforator “branch steal,” we considered hypoperfusion in septal/anterior segments immediately adjacent to the tunneled portion of coronary artery as functionally related to the MB. Quantitative analysis focused on subendocardial regions of interest corresponding to at least 0.5 cm^3^ of myocardium. MBF was measured by two cardiovascular radiologists, each with more than 10 years of experience, blinded to clinical and anatomical data. In the event of disagreement, a consensus reading was used to determine the final interpretation.

Following validated criteria, patients were categorized into two groups based on perfusion findings in the MB territory. The hypoperfusion group included patients presenting with at least one segment within the MB-related perfusion territory showing an MBF ratio ≤ 0.85, relative to remote reference segments. Conversely, patients without any segmental perfusion defect in the MB-related were assigned to the no hypoperfusion group.

### 2.5. Statistical Analysis

Statistical analysis was performed by the statistical software MedCalc (MedCalc Software, 14.8.1 version, Bvba, Groot-Bijgaarden, Belgium). Continuous variables were expressed as mean ± standard deviation or as median and interquartile range, depending on the distribution assessed by the Shapiro–Wilk test. Categorical variables were presented as absolute numbers and percentages. Comparisons between patients with and without myocardial hypoperfusion were performed using the Mann–Whitney U test for continuous variables, and the chi-square test for categorical variables. A multivariable logistic regression analysis was conducted to identify independent predictors of myocardial hypoperfusion. Given the relatively low number of events, we limited the number of covariates to maintain an adequate events-per-variable ratio. Clinical and global atherosclerosis indices were excluded from the regression model to avoid model overfitting given the event size. In addition, penalized regression methods were tested in sensitivity analyses, which confirmed the robustness of the main findings. To evaluate the diagnostic performance of bridge depth in predicting myocardial hypoperfusion, a receiver operating characteristic (ROC) curve analysis was performed. The area under the curve (AUC) was calculated to assess the overall discriminative ability. The optimal cut-off value for bridge depth was identified using Youden’s index (sensitivity + specificity – 1), and corresponding sensitivity and specificity values were reported. AUC values between 0.7 and 0.8 were considered acceptable, and values ≥ 0.8 were considered excellent. For each acquisition phase, we recorded the dose-length product (DLP) and calculated the effective dose (ED) using a conversion factor of 0.014 mSv/mGy·cm. A *p*-value < 0.05 was considered statistically significant.

## 3. Results

### 3.1. Study Population

Of the 81 patients included in the final analysis, the majority were male (66.7%), with a mean age of 59.3 ± 11.7 years (normal distribution, *p* = 0.71). Mean BMI was 23.1 ± 2.3 kg/m^2^ (normal distribution, *p* = 0.30). There were no significant differences between the hypoperfusion and no-hypoperfusion groups in terms of age, sex distribution, body mass index and cardio-vascular risk factors. Resting and stress ECG abnormalities suggestive of ischemia were more frequently reported in the hypoperfusion group, although these differences did not reach statistical significance (Table 1). Mean DLP was 40.8 ± 13.5 mGy·cm for the non-contrast scan, 149.1 ± 46.8 mGy·cm for CCTA, and 253.7 ± 60.3 mGy·cm for stress dynamic CT-MPI. The mean radiation dose, expressed as the ED, was 6.2 ± 1.3 mSv.

### 3.2. CCTA and Stress Dynamic CTP Analysis

All MBs were located in either the mid or distal segment of the left anterior descending artery (LAD), with 42 cases (51.9%) in the mid-LAD and 39 (48.1%) in the distal-LAD. Myocardial hypoperfusion in the vascular territory supplied by the bridged segment was observed in 26 patients (32.1%), while the remaining 55 patients (67.9%) showed no perfusion abnormalities. Interobserver agreement for perfusion defect detection was excellent (κ = 0.86, 95% CI: 0.78–0.94). In 6/81 patients (7.4%), initial disagreement occurred, which was resolved by consensus. These adjudications did not alter the overall classification of patients into hypoperfusion vs. no-hypoperfusion groups.

**Figure 1:**
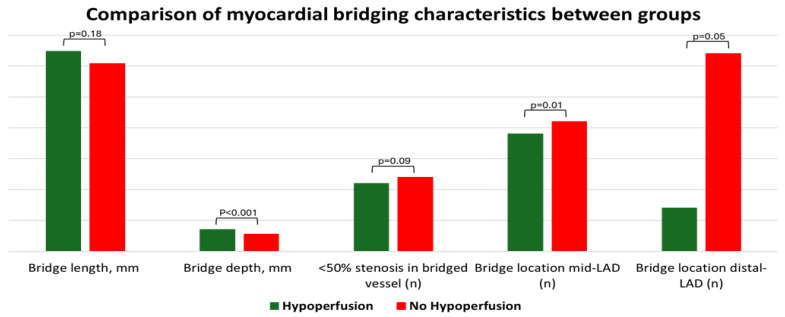
Anatomical Features of Myocardial Bridging on Coronary CTA.

The median Agatston CACS was higher in the hypoperfusion group compared to those without, although this difference did not reach statistical significance (*p* = 0.0911). The distribution of CAD-RADS categories (0, 1, and 2) was similar across both groups, with no significant differences observed (all *p* > 0.7). The average EAT volume was slightly higher in patients with hypoperfusion, but this difference was not statistically significant (*p* = 0.2731). Among anatomical characteristics of MBs, bridge depth was significantly greater in patients with hypoperfusion (*p* < 0.001), while bridge length showed a non-significant trend toward higher values in the same group (*p* = 0.1824). A non-obstructive stenosis (<50%) in the bridged vessel was more frequently observed in the hypoperfusion group (42.3% vs. 21.8%, *p* = 0.0992), though this did not reach significance. MBs located in the mid-LAD were significantly more common among patients with hypoperfusion, whereas bridges in the distal-LAD were more frequent among patients without hypoperfusion (58.2% vs. 26.9%, *p* = 0.0511), though this did not reach significance (Table 2). Figure 1 illustrates these data.

**Figure 2:**
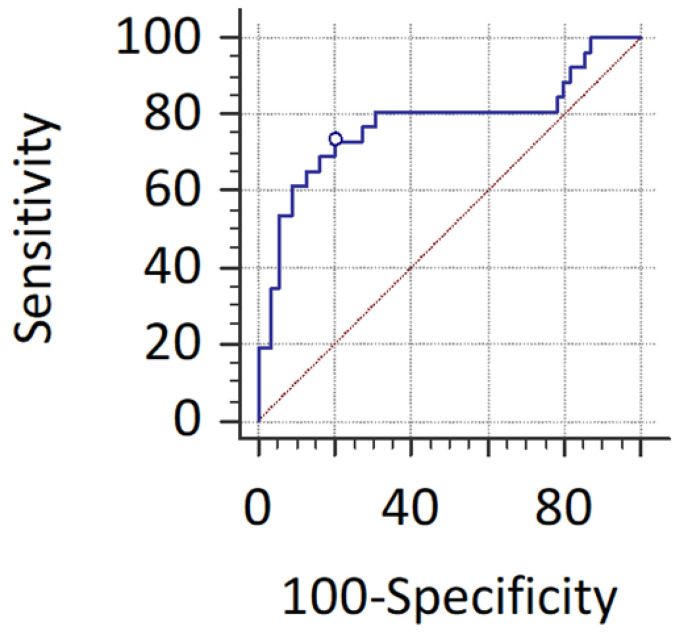
ROC curve analysis of myocardial bridge depth. The image show the ROC curve analysis demonstrated that bridge depth had a discriminative power for detecting myocardial hypoperfusion (AUC of 0.77 ± 0.06, <0.001). The optimal cut-off value, based on Youden’s index, was 3.26 mm (blue circle), yielding a sensitivity of 73.1% and a specificity of 80.0%.

**Figure 3:**
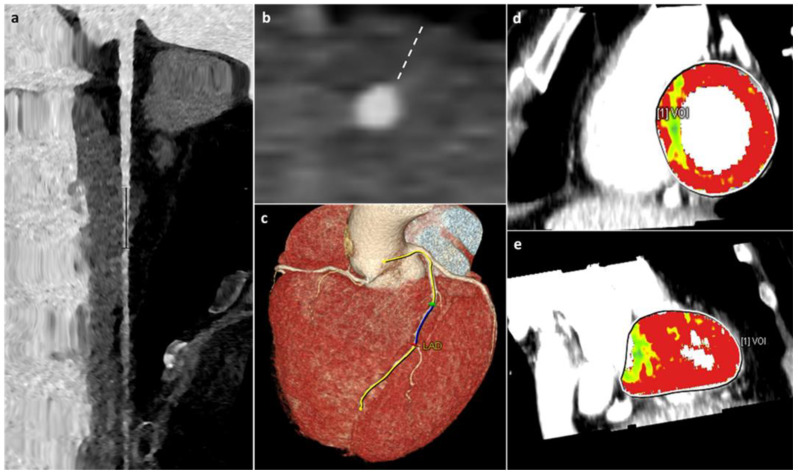
A 56-year-old male patient with a myocardial bridge on the mid-LAD. The image shows the coronary CT assessment of a myocardial bridge. (**a**) Curved multiplanar reconstruction (MPR) showing a myocardial bridge on the mid-LAD extending for approximately 27 mm (black line). (**b**) Cross-sectional view demonstrating the depth of the myocardial bridge, measured at 2.8 mm (dot white line). (**c**) Volume-rendered 3D image of the coronary tree, with the course of the mid-LAD highlighted; the tunneled segment (blue) is embedded within the myocardium. (**d**,**e**). Stress myocardial perfusion maps in short-axis (**d**) and vertical long-axis (**e**) views show a corresponding area of hypoperfusion (green) in the basal septum, consistent with the functional significance of the myocardial bridge.

**Figure 4:**
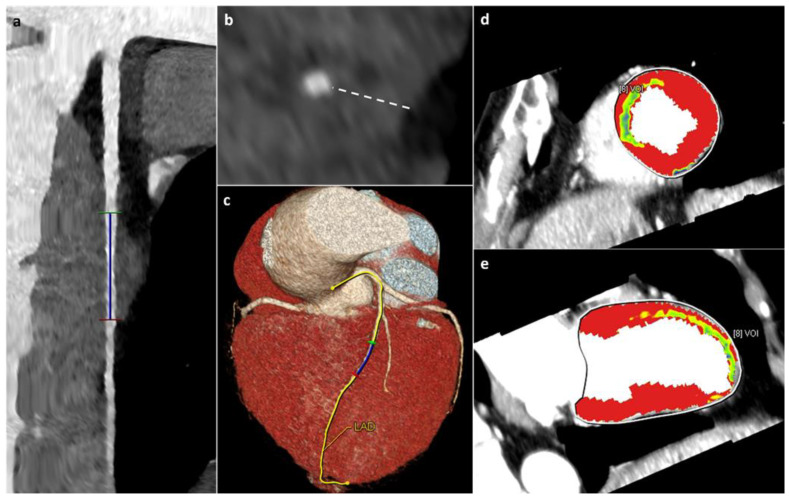
A 61-year-old female patient with a myocardial bridge on the distal-LAD. The image shows the coronary CT assessment of a myocardial bridge in the distal-LAD with functional correlation. (**a**) Curved multiplanar reconstruction (MPR) demonstrates a myocardial bridge extending for approximately 24 mm in the distal-LAD (blue line). (**b**) Cross-sectional image shows the depth of the bridge embedded in the myocardium, measured at 4.1 mm (dot white line). (**c**) Volume-rendered 3D reconstruction highlights the course of the LAD, with the tunneled segment (blue) clearly embedded in the myocardial tissue. (**d**,**e**) Stress CT myocardial perfusion maps in short-axis (**d**) and vertical long-axis (**e**) views demonstrate a matching area of hypoperfusion (green) in the anteroseptal mid-to-apical segments, consistent with the functional significance of the myocardial bridge.

**Figure 5:**
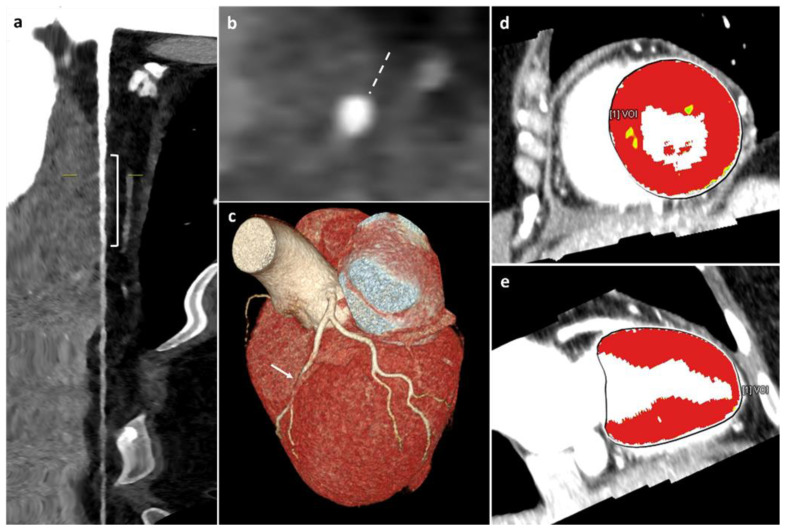
**A 58-year-old male patient with a myocardial bridge on the distal-LAD.** The image shows the coronary CT assessment of a myocardial bridge in the mid-LAD. (**a**) Curved multiplanar reconstruction (MPR) demonstrates a myocardial bridge extending for approximately 34 mm in the distal-LAD (white line). (**b**) Cross-sectional image shows the depth of the bridge embedded in the myocardium, measured at 2.1 mm (dot white line). (**c**) Volume-rendered 3D reconstruction highlights the course of the LAD, with the tunneled segment (white arrow) clearly embedded in the myocardial tissue. (**d**,**e**) Stress CT myocardial perfusion maps in short-axis (**d**) and vertical long-axis (**e**) views does not demonstrate a corresponding area of hypoperfusion.

A multivariable logistic regression model was constructed with MBs variables. Among these, bridge depth and mid-LAD location remained independently associated with myocardial hypoperfusion (Table 3). ROC curve analysis demonstrated that bridge depth had a discriminative power for detecting myocardial hypoperfusion, with an AUC of 0.77 ± 0.06 (IC 95%: 0.671–0.862; *p* < 0.001). The optimal cut-off for MB depth was derived de novo using Youden’s index from ROC analysis. The depth threshold of 3.26 mm yielded a sensitivity of 73.1% (95% CI: 52.2–88.4%) and specificity of 80.0% (95% CI: 66.3–90.0%) (Figure 2). Figure 3, Figure 4 and Figure 5 illustrate representative cases from our study population, highlighting anatomical and functional findings of MB in individual patients.

## 4. Discussion

MB is a common congenital variant, often detected incidentally on CCTA. While typically benign, certain anatomical features may confer functional significance, potentially leading to ischemia. Traditional functional tests, such as SPECT or invasive FFR, have inherent limitations. Recent advances in CT technology now enable combined anatomical and perfusion assessment using dynamic CT-MPI [1,2,3]. The aim of this study was to evaluate whether specific CT characteristics of MBs with high-risk anatomical features are independently associated with myocardial hypoperfusion, as assessed by stress dynamic CT-MPI.

To our knowledge, this is the first study to specifically investigate MBs with high-risk characteristics (length ≥ 25 mm and a depth ≥ 2.0 mm) using a combined anatomical and functional CT-based approach.

Our findings demonstrate that bridge depth is independently associated with perfusion abnormalities, supporting the notion that MB is not merely an anatomical variant, but can be a hemodynamically significant condition in selected patients [3]. The presence of myocardial hypoperfusion in patients with deep MBs may be explained by multiple interrelated physiological mechanisms. Deeper bridges are more likely to induce marked and prolonged systolic compression that can extend into early diastole, reducing the effective time window for coronary perfusion. Moreover, increased intramyocardial pressure associated with bridge depth may create a higher transmural pressure gradient, further compromising subendocardial perfusion. In addition, altered local hemodynamics, including turbulence within the bridged segment, may reduce distal perfusion pressure [16]. Chronic abnormal shear stress at the site of the bridge may also lead to endothelial dysfunction and contribute to impaired microvascular regulation [17]. Another potential pathophysiological mechanism is the phenomenon of “branch steal,” particularly involving septal perforator arteries. During late systole and early diastole, blood passing through the compressed segment of the bridged artery accelerates due to luminal narrowing, which may reduce perfusion pressure at the origin of adjacent branches—such as septal arteries—via the Venturi effect. This mechanism could contribute to regional hypoperfusion despite the absence of significant epicardial coronary stenosis [16]. Consistent with the present results, Zhao et al. [15] highlighted the role of bridge depth as a key determinant of myocardial hypoperfusion. Similarly, Yu et al. [18] demonstrated a significant association between bridge depth and myocardial ischemia, underscoring its central role among the anatomical predictors of functional impairment in MB.

Our findings indicate a significant association between myocardial hypoperfusion and MBs located in the mid-segment of the LAD. Several anatomical and physiological factors may explain this observation. The mid-LAD typically supplies a larger portion of the anterior and anteroseptal myocardial wall—regions with high metabolic demand and limited collateral circulation. Consequently, even subtle reductions in coronary flow caused by bridging in this segment may result in clinically relevant perfusion defects. This anatomical configuration may increase the likelihood of delayed diastolic relaxation and impaired coronary filling. Furthermore, the mid-LAD traverses a thicker myocardial territory compared with distal segments, which could amplify extrinsic compressive forces during systole and early diastole. These findings highlight the importance of not only identifying the presence of an MB but also considering its anatomical location when assessing the risk of functional impairment.

Our findings are consistent with contemporary literature suggesting that bridge length alone is not a reliable indicator of hemodynamic significance. These findings reinforce the concept that a long but shallow or superficially located MB may have minimal functional impact, whereas a relatively short but deep bridge may carry a higher ischemic risk. In line with Zhao et al. [15], bridge length was not significantly associated with myocardial ischemia.

Furthermore, no significant differences in clinical characteristics were found between patients with and without hypoperfusion. In our cohort, the prevalence of angina was comparable in patients with and without hypoperfusion, suggesting that clinical symptoms alone may not reliably indicate the functional significance of MB. This finding is consistent with the observations of Gannon et al. [14] emphasizing the limited diagnostic accuracy of symptoms in this setting and highlights the incremental value of CT perfusion imaging, which provides an objective assessment of downstream myocardial blood flow beyond purely anatomical or clinical evaluation.

Another contributing factor is the frequent coexistence of a proximal non-obstructive coronary stenosis, which may act synergistically with the bridge to produce a “double resistance” effect, further reducing flow reserve. Doppler studies have shown retrograde flow proximal to the bridge during systole, which may disrupt normal antegrade flow. This mechanism is thought to contribute to the preferential development of atherosclerotic plaque just proximal to the bridged segment [19,20]. However, in our study, the presence of <50% stenosis in the vessel segment containing the MB was not significantly associated with myocardial hypoperfusion. Several hypotheses may account for this finding. First, stenosis assessment by CCTA in dynamically compressed segments is technically challenging and may occasionally overestimate its severity. In addition, in deep MBs, the tunneled segment itself may represent the main source of flow resistance, thereby reducing the incremental impact of mild proximal plaques. In contrast to our results, a previous study evaluated myocardial perfusion abnormalities using SPECT in patients with MB but included only cases with concomitant stenosis greater than 50% in the affected artery [21].

Although patients with hypoperfusion tended to have higher CACS, more advanced CAD-RADS categories, and a slightly greater EAT volume, none of these differences reached statistical significance in our cohort. The lack of statistical significance may be due to the relatively small sample size and the exclusion of patients with obstructive CAD, which limited the overall variability in the study population.

Finally, recent evidence by Yu et al. [18] suggests that CT-FFR may serve as a valuable adjunct in assessing the functional significance of MB, showing high sensitivity and negative predictive value for detecting ischemia. Although not explored in our study, this parameter could be integrated in future analyses to enhance the diagnostic accuracy of non-invasive evaluation.

The limitations of this study include the relatively small sample size, which may reduce the statistical power to detect weaker associations, and its retrospective design, which inherently carries the risk of selection bias and limits the ability to establish causal relationships. Furthermore, invasive hemodynamic measurements, such as fractional flow reserve or intravascular ultrasound, were not performed, which would have provided a direct physiological validation of our findings.

## 5. Conclusions

In conclusion, our findings suggest that MBs with specific anatomical features, namely a greater depth and proximal location in the mid-LAD, may be more frequently associated with myocardial hypoperfusion. While these results support the potential value of a combined anatomical and functional CT approach, they should be interpreted with caution given the retrospective design and limited sample size. This combined approach may improve risk stratification and help identify patients who may benefit from closer monitoring or targeted therapy. Larger, prospective studies are warranted to validate these preliminary observations and to better define the clinical implications of MB-related perfusion abnormalities.

## Figures and Tables

**Table 1:** Clinical Characteristics of the Study Population.

Variable	Total (*n* = 81)	Hypoperfusion (*n* = 26)	No Hypoperfusion (*n* = 55)	*p*-Value
Age, years (mean ± SD)	59.3 ± 11.7	61.8 ± 10.5	57.5 ± 12.1	0.1239
Male sex (*n*, %)	54 (66.7%)	17 (65.4%)	37 (67.3%)	0.9410
BMI, kg/m^2^ (mean ± SD)	23.1 ± 2.3	23.6 ± 2.1	22.8 ± 2.4	0.5867
Diabetes mellitus (*n*, %)	11 (13.6%)	4 (15.4%)	7 (12.7%)	0.8148
Hypertension (*n*, %)	43 (53.1%)	13 (50.0%)	30 (54.5%)	0.8381
Dyslipidemia (*n*, %)	30 (37.0%)	11 (42.3%)	19 (34.5%)	0.6391
Current smoking (*n*, %)	16 (19.8%)	6 (23.1%)	10 (18.2%)	0.7753
Angina symptoms, *n* (%)	56 (69.1%)	17 (65.4%)	39 (70.9%)	0.9241
Rest ECG ischemic abnormalities (*n*, %)	15 (18.5%)	6 (23.1%)	9 (16.4%)	0.6759
Exercise ECG ischemic abnormalities (*n*, %)	23 (28.4%)	12 (46.2%)	14 (25.5%)	0.2071

Abbreviations—SD: standard deviation; BMI: body mass index; ECG: electrocardiogram.

**Table 2:** Anatomical Features of Myocardial Bridging on Coronary CTA.

Variable	Total (*n* = 81)	Hypoperfusion (*n* = 26)	No Hypoperfusion (*n* = 55)	*p*-Value
Calcium score, Agatston (median, IQR)	122 [180]	158 [180]	92 [160]	0.0911
CAD-RADS 0 (*n*, %)	20 (24.7%)	6 (23.1%)	14 (25.5%)	0.9713
CAD-RADS 1 (*n*, %)	34 (42.0%)	13 (50.0%)	21 (47.3%)	0.7186
CAD-RADS 2 (*n*, %)	23 (28.4%)	7 (26.9%)	15 (27.3%)	0.8221
EAT volume (cm^3^) (median, IQR)	92.9 [35.1]	93.5 [37.4]	91.4 [34.6]	0.2731
Bridge length, mm (median, IQR)	32.1 [7.9]	33.9 [10.8]	29.9 [7.4]	0.1824
Bridge depth, mm (median, IQR)	3.0 [0.9]	3.5 [0.8]	2.9 [0.9]	<0.001
<50% stenosis in bridged vessel (*n*, %)	23 (28.4%)	11 (42.3%)	12 (21.8%)	0.0992
Bridge location mid-LAD (*n*, %)	42 (51.9%)	19 (73.1%)	21 (38.2%)	0.0181
Bridge location distal-LAD (*n*, %)	39 (48.1%)	7 (26.9%)	32 (58.2%)	0.0511

Abbreviations—IQR: interquartile range; CAD-RADS: coronary artery disease-reporting and data system; EAT: epicardial adipose tissue; LAD: left anterior descending artery; SD: standard deviation.

**Table 3:** Multivariable Logistic Regression Analysis for Predictors of Myocardial Hypoperfusion.

Variable	Coefficient	Standard Deviation	*p*-Value
Bridge length	0.01	0.09	0.0953
Bridge depth	0.41	0.08	<0.001
<50% stenosis in bridged vessel	0.11	0.01	0.2512
Bridge location mid-LAD	0.23	0.09	0.0231
Bridge location distal-LAD	−0.13	0.09	0.1691

Abbreviations—IQR: interquartile range; CAD-RADS: coronary artery disease-reporting and data system; EAT. Epicardial adipose tissue; LAD: left anterior descending artery; SD: standard deviation.

## Data Availability

The data presented in this study are available on request from the corresponding author.
